# A mechanism-based group psychotherapy approach to aggressive behavior (MAAP) in borderline personality disorder: a multicenter randomized controlled clinical trial

**DOI:** 10.1186/s13063-025-08985-6

**Published:** 2025-08-02

**Authors:** Christine Sigrist, Andreas Bechdolf, Katja Bertsch, Robin Bullenkamp, Martin Busse, Ute gr. Darrelmann, Astrid Dempfle, Martin Driessen, Thomas Frodl, Jan-Michael Kersting, Jessica Kesik, Burkhart Matzke, Corinne Neukel, Eva Niessen, Svenja Nückel, Viola Oertel, Frank Padberg, Alexandra Philipsen, Dorothee Pink, Andreas Reif, Matthias Reinhard, Carolin Steuwe, Larissa Wolkenstein, Sabine C. Herpertz, the MAAP Consortium

**Affiliations:** 1 Department of General Psychiatry, Heidelberg University, Heidelberg, Germany; 2 Department of Psychiatry, Psychotherapy and Psychosomatics, Vivantes Klinikum Am Urban and Vivantes Klinikum Im Friedrichshain, Berlin, Germany; 3 Department of Psychiatry and Psychotherapy, CCM, Charité Universitätsmedizin Berlin, corporate member of, FreieUniversität Berlin and Humboldt-University of Berlin , Berlin, Germany; 4 DZPG (German Center for Mental Health), Partner Site Berlin-Potsdam, Berlin, Germany; 5 Department of Psychology, Julius-Maximilians-Universität Würzburg, Würzburg, Germany; 6 Center for Mental Health, Gesundheitszentrum Odenwaldkreis GmbH, Erbach, Germany; 7 Department of Psychiatry and Psychotherapy, University Hospital Bonn, Bonn, Germany; 8 Institute of Medical Informatics and Statistics, Christian-Albrechts University of Kiel, Kiel, Germany; 9 Department of Psychiatry and Psychotherapy, Evangelisches Klinikum Bethel, Bielefeld, Germany; 10 Department of Psychiatry and Psychotherapy, University Hospital Aachen, RWTH, Aachen, Germany; 11 Department of Psychiatry and Psychotherapy, Carl-Friedrich-Flemming-Klinik (CFFK), Helios Kliniken Schwerin, Schwerin, Germany; 12 Department of Psychiatry and Psychotherapy, University Hospital LMU, Munich, Germany; 13 Department of Psychiatry, Psychosomatic Medicine and Psychotherapy, University Hospital Frankfurt, Frankfurt Am Main, Germany; 14 Department of Psychology, LMU, Munich, Germany

**Keywords:** Anger, Reactive aggression, Aggressive behavior, Borderline personality disorder, Group psychotherapy, Dialectic behavior therapy, Mentalization-based therapy, Mindfulness, Emotion regulation, Impulsivity

## Abstract

**Background:**

High levels of trait anger and aggressive behavior are common and problematic phenomena in patients with borderline personality disorder (BPD). In BPD, patterns of reactive aggression often lead to functional impairment affecting important areas of life. Despite the high burden on individuals and their social environment, there are no specific, cost-effective treatments to reduce aggression in BPD. In previous studies, we and others have been able to infer specific biobehavioral mechanisms underlying patterns of reactive aggression in BPD that can be used as potential treatment targets. To address this, we developed a mechanism-based anti-aggression psychotherapy (MAAP) for the group setting that specifically targets the biobehavioral mechanisms underlying outward-directed aggression in BPD. A previously conducted proof-of-concept study had suggested beneficial effects for this neglected group of patients.

**Methods:**

In this multicenter, confirmatory, randomized-controlled-clinical-trial, MAAP, which consists of multifaceted, evidence-based treatment elements adapted from other sophisticated treatment programs such as Dialectical Behavior Therapy and Mentalization-Based Treatment, is tested for efficacy against a non-specific supportive psychotherapy (NSSP) program focusing on non-specific general factors of psychotherapy at seven different sites in Germany. Both treatment arms, based on one individual and 13 group therapeutic sessions (1.5 h per session, twice a week), are delivered over a period of 7–10 weeks. A total of *N* = 186 patients will be recruited, half of whom will be cluster-randomized to MAAP. Outcomes are assessed at baseline, immediately, and 4, 12, 20, and 24 weeks post-treatment using ecological momentary assessment, clinical interviews, questionnaires, and online tasks.

**Discussion:**

If proven superior, MAAP can be incorporated into standard psychiatric care, filling a critical gap in the current therapeutic landscape by offering a structured, cost-effective, and evidence-based treatment that directly targets the biobehavioral mechanisms underlying reactive aggression in BPD. By potentially improving clinical outcomes and reducing the burden of reactive aggression in BPD, MAAP could be beneficial for both individuals and their social environments. The study’s large, multicenter design enhances the generalizability of the results, making them more relevant for broader clinical applications.

**Trial registration:**

This study was registered in the German Clinical Trials Register DRKS (DRKS00031608) on 31.10.2023 (https://drks.de/search/de/trial/DRKS00031608).

## Introduction

### Background and rationale {6a}

Borderline personality disorder (BPD) is a common and severe debilitating mental disorder that affects approximately 1.7% of the general population and up to 28% of inpatients [1]. BPD is characterized by persistent patterns of instability in affect regulation, impulse control, interpersonal relationships, and self-image [2]. It is also marked by a strong tendency toward feelings of anger and aggression, which further represents one of nine criteria for diagnosing BPD according to standard diagnostic classification systems (ICD, DSM). Critically, reactive aggressive behavior toward others, usually triggered by real or perceived interpersonal threat, rejection, and provocation, is a very common phenomenon and represents a major burden in both female and male patients with BPD [3–5]. While in fact a large proportion of patients engage in self-harming and self-injurious behaviors [6], up to 80% of patients with BPD also report engaging in aggressive behavior toward others during the course of a year [3, 7]. Yet, the majority of available studies and interventions for BPD focus on aggressive behaviors directed towards the self, and despite the substantial burden on individuals and their social environment, there are no specific, cost-effective treatment options to reduce (reactive) aggressive behavior in BPD, indicating a critical research and treatment gap in this field.

Based on the assumption that treatment development must be tailored to the pathogenic mechanisms associated with a particular psychopathology [8, 9], to address this research and treatment gap, we developed a mechanism-based anti-aggression psychotherapy (MAAP) for the group setting that specifically targets outward-directed aggression in BPD. We designed this program to target mechanisms that we have identified as underlying reactive aggression in BPD and as summarized in a model previously proposed by our group [10]. First, social threat hypersensitivity, i.e., hypervigilance to social threat cues, biased negative perceptions of others, and an inability to recognize social safety signals (for an overview, see [11]); second, approaching social threat cues rather than avoiding them (threat approach rather than avoidance) [12]; third, maladaptive anger regulation (for an overview, see [13]), with emotion dysregulation and anger sequentially mediating the relationship between BPD and aggression [14]; fourth, excessive emotional imitation and contagion (for an overview see [15]); fifth, difficulty mentalizing others’ intentions, cognitions, and emotions adequately (for an overview see [16, 17]). These mechanisms are assumed to interact with each other, e.g., there is a close relationship between social threat hypersensitivity and anger [18], and between low mentalizing ability and social threat hypersensitivity [19] and poor anger regulation [20]. Thus, what makes this group therapeutic approach “mechanism-based” is the targeted focus on specific biobehavioral mechanisms. Unlike more general approaches, such as Dialectical Behavior Therapy (DBT) or Mentalization-Based Treatment (MBT), which focus on broader emotional regulation or mentalizing difficulties—and which have shown some promising effects in reducing aggression and anger in BPD (i.e. [21–24],)—MAAP is explicitly designed to address these distinct mechanisms contributing to (reactive) aggressive behavior toward others. For instance, while DBT emphasizes emotional regulation and distress tolerance, and MBT targets improving mentalizing capacities, MAAP combines these elements but integrates specific attention training techniques (such as an app-based training to reduce threat hypersensitivity) and aims to specifically target maladaptive social perception and behavior patterns that contribute to aggression. In addition, the interventions included in MAAP are designed to target identified mechanisms while remaining focused and allowing for high frequency of repetition within a short, low-cost intervention (see the Interventions section for more details on MAAP) [25].

Based on our proof-of-concept study [25], in which we found a clinically relevant reduction in aggressive behavior and significant differences in the reduction of overt aggression between MAAP and control treatment at follow-up, the present trial aims to investigate the efficacy of MAAP in a larger multicenter, confirmatory, randomized-controlled trial (RCT). In case of superiority, the time-limited group program can likely be successfully implemented in psychiatric care.

## Objectives {7}

The primary objective of this trial relates to the hypothesis of superiority of MAAP over a control intervention (i.e., non-specific supportive psychotherapy, NSSP) [26], which is supported by a pilot study of our research group [25]. The primary hypothesis is that patients in the MAAP condition, compared with patients in the NSSP condition, will show a significant reduction in overt aggressive behavior, as measured by the Overt Aggression Scale Modified (OAS-M) [27], 24 weeks after the end of the intervention at follow-up (T5) compared with baseline (T0).

The secondary objectives, leading to eight secondary hypotheses, include further indicators of the hypothesized superiority of MAAP over NSSP. First, it is hypothesized that patients in the MAAP condition will show a significantly greater reduction in overt aggressive behavior (OAS-M) immediately after the intervention (T1) and/or at follow-up measurements 4 weeks (T2), 12 weeks (T3), and 20 weeks (T4) after the intervention compared to patients in the NSSP condition and as measured at baseline. Second, it is hypothesized that patients in the MAAP condition will show a significantly greater reduction in auto-aggressive behavior (OAS-M) and/or irritability (OAS-M) at T1 and/or at follow-up measurements T2–T5 compared to patients in the NSSP condition and as measured at baseline. Third, it is hypothesized that patients in the MAAP condition will show a significantly higher response rate (with response being defined as reduction of the OAS-M overt aggression score by at least 50% from baseline) at T1 and at all follow-up measurements (T2–T5) compared to patients in the NSSP condition. Fourth, it is hypothesized that after 24 weeks of follow-up, patients in the MAAP condition will show a significantly greater reduction in anger experience (STAXI-II) and aggressive behavior (3 additional items), as measured via EMA (8 assessments/day) over 2 weeks, than patients in the NSSP condition and as measured at baseline. Fifth, it is hypothesized that patients in the MAAP condition will show a significantly greater reduction in BPD symptom severity (ZAN-BPD) at T1, T3, and at T5 compared to patients in the NSSP condition and as measured at baseline. Sixth, it is hypothesized that patients in the MAAP condition will show a significantly greater reduction in trait anger (STAXI-II) at T1, T4, and T5 compared to patients in the NSSP condition and as measured at baseline. Seventh, it is hypothesized that patients in the MAAP condition will show a significantly greater improvement in everyday functioning (SOFAS) at T1, T2, and T5 compared to patients in the NSSP condition and as measured at baseline. Eighth, it is hypothesized that patients in the MAAP condition will show a significantly greater improvement in quality of life (WHO-QoL-Bref) at T1, T3, and T5 compared to patients in the NSSP condition and as measured at baseline.

Of note, while the secondary hypotheses aim to further support the primary hypothesis of the superiority of MAAP over NSSP, they will be tested independently, and no formal correction for multiple comparisons will be applied. Given the exploratory nature of the secondary objectives and the number of comparisons across multiple outcome domains, we acknowledge the increased risk of type I error. Therefore, results from secondary analyses will be interpreted cautiously, with emphasis placed on the consistency and robustness of findings across outcomes. All p-values will be reported alongside corresponding effect sizes and confidence intervals to support transparent interpretation. This approach aims to balance the risk of false positives with the exploratory purpose of these analyses in informing future confirmatory research.

In addition to primary and secondary outcomes, the study includes exploratory outcome measures (i.e., questionnaire and behavioral data) that assess additional constructs relevant to BPD, anger regulation, and mechanism-based therapeutic approaches. These outcomes are less directly related to the main research objective but are nonetheless meaningful in the current clinical and theoretical context. Exploratory analyses will also include mediation and moderation models to investigate potential mechanisms of change. These analyses are primarily hypothesis-generating and will be interpreted conservatively, taking into account their broader scope and less stringent pre-analysis specification.

All outcomes of the study (primary, secondary, and exploratory) are listed below in the section on *Outcomes*.

## Trial design {8}

The study is an RCT that is currently ongoing and is designed to evaluate the efficacy of a specific treatment intervention. It applies a parallel group design with two treatment arms, one receiving the experimental intervention and the other receiving an active control treatment. The primary objective of the trial is to assess the superiority of the experimental treatment over the active control. The trial follows an interventional and confirmatory design to test the efficacy of the treatment under investigation. Participants are randomly assigned to one of the two treatment arms by central randomization, stratified by center. Randomization is performed at the treatment group level, with each group consisting of usually 4 to 6 patients, using cluster randomization. The trial is blinded, with the outcome assessors unaware of the group assignments to reduce potential bias. Sequences are generated using computer-generated random numbers to ensure unbiased allocation of participants.

## Methods: participants, interventions, and outcomes

### Study setting {9}

This is a multicenter study conducted at several study sites in Germany. Recruitment is taking place at various university hospitals and research institutions specializing in psychiatry and psychotherapy. Further details of the participating sites can be found on the study registration page (https://drks.de/search/de/trial/DRKS00031608).

### Eligibility criteria {10}

Inclusion criteria for participants are.

- Minimum age 18 years
- Maximum age 60 years
- Outpatients with at least four diagnostic criteria of BPD fulfilled according to the structured clinical interview International Personality Disorder Examination (IPDE based on DSM-5) [28].
- Overt aggression based on OAS-M (sum of items 1–3) ≥ 15 and irritability based on OAS-M (sum of items 5–6) ≥ 6 within two weeks prior to study inclusion
- Living within 70 km of a study center or within easy reach of a study center
- Sufficient knowledge of German for participation in group therapy

Exclusion criteria for participants are.

- Suicidality (assessed on an individual basis by a clinician after positive screening on OAS-M)
- Acute/previous schizophrenia (assessed on an individual basis by a clinician after positive screening on MINI-DIPS)
- Acute bipolar I disorder assessed on an individual basis by a clinician after positive screening on MINI-DIPS)
- Acute moderate to severe substance use disorder
- Autism Spectrum Disorder assessed on an individual basis by a clinician after positive screening on AQ-10
- No ability or willingness to abstain from drug or alcohol use during therapy sessions (assessed by a clinician on an individual basis)
- History of a serious medical condition that interferes with participation in regular therapy sessions
- Severe cognitive impairment (IQ < 70) assessed on an individual basis by a clinician after positive screening on mini-q
- Other ongoing psychotherapy
- Increase in current psychotropic medication or initiation of new psychotropic medication within two weeks prior to study assignment (6 weeks instead of 2 weeks for fluoxetine)
- Ongoing criminal investigation, placement in a forensic hospital or incarceration for more than 2 years

### Therapists

All therapists in this study are qualified psychologists or psychiatrists in advanced training (at least 2 years) with experience in working with patients with BPD. Participating therapists are trained prior to delivering the study treatment and are required to deliver both treatment conditions in order to reduce effects of specific therapists. Training to deliver the study treatment comprises a) virtual and on-site training and b) piloting in both treatment conditions (NSSP and MAAP). Piloting is an abbreviated form of trial therapy in which the therapies learned in training are tested under regular supervision. In sites where experienced trial therapists are already in place and new therapists have undergone step (a) training, they may as an alternative to step (b) accompany experienced therapists in trial therapy in each condition. In this case, piloting is no longer necessary.

### Who will take informed consent? {26a}

Each participant receives a detailed description of the study from the diagnostician-in-charge (psychologist with at least a Master's degree) and signs an informed consent form approved by each local ethics committee in the presence of the diagnostician indicating their willingness to participate in the clinical trial. Informed consent is obtained at each site immediately at the beginning of the diagnostic enrollment session, before the inclusion and exclusion criteria are assessed during a detailed diagnostic evaluation.

### Additional consent provisions for collection and use of participant data and biological specimens {26b}

Not applicable. The informed consent covers all necessary information.

## Interventions

### Explanation for the choice of comparators {6b}

The chosen control condition (NSSP) is implemented as an active control intervention offered in the same setting and dosage as MAAP (i.e., same number of participants and therapists, same frequency and duration of each session, same duration of training, piloting, number of supervisions, etc.). NSSP (see section below) offers advantages over treatment as usual (TAU) or waitlist control conditions and has been used as a control intervention for related therapies such as Dialectical Behavior Therapy [29]. This approach ensures that the effects on the target criteria are attributable to the specific psychotherapeutic program.

### Intervention description {11a}

The experimental treatment (MAAP) consists of various elements drawn from sophisticated evidence-based psychotherapy programs, such as Dialectical Behavior Therapy [30] and Mentalization-Based Treatment [24]. Experts by experience contributed to the selection of interventions and safety issues. To these multifaceted evidence-based elements, we added specific attention training techniques, i.e., an app-based attention training delivered between sessions that directs attention to safe instead of threatening social cues and, thus, counteracts threat hypervigilance. The elements were combined to specifically target the hypothesized mechanisms underlying reactive aggression in BPD, that is, social threat hypersensitivity, approaching social threat cues rather than avoiding them, maladaptive anger regulation, excessive emotional imitation and contagion, and difficulty mentalizing others’ intentions, cognitions, and emotions adequately [15]. This therapy is delivered in a group setting (clusters of 4–6 patients are formed and randomized, if necessary for organizational reasons, 3 or 7 patients will also be allowed) and includes two 90-min group sessions per week, as well as one individual preparatory session. In total, each participant receives 14 sessions (1 individual and 13 group sessions) delivered over a period of 7–10 weeks. The extended time frame allows for flexibility in scheduling, including the possibility of rescheduling missed sessions due to therapist absence, holidays, or other disruptions.

**Fig. 1:**
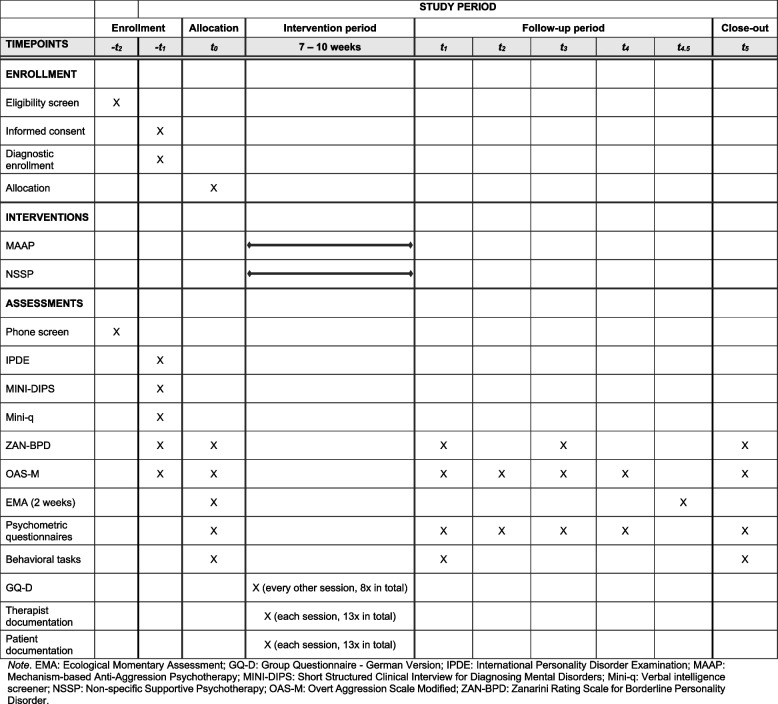
Study schedule for enrollment, interventions, and assessments

The control intervention (NSSP) is based on an adapted treatment manual [26] developed to address non-specific therapeutic factors. Thus, NSSP focuses on non-specific, “general” factors of psychotherapy, including psychoeducational elements and a therapeutic stance characterized by reflective listening, empathy, therapeutic optimism, and recognition and promotion of the patient’s resources. This therapeutic stance creates a stable, explicitly supportive therapeutic relationship. NSSP does not teach specific skills and does not include specific cognitive, behavioral, or psychodynamic interventions. The manual was supplemented with recommendations for dealing with critical situations that may arise in the group setting with patients prone to aggressive behavior (e.g., highly affectively charged situations, aggressive outbursts, suicidality, dissociative states, oppositional behavior) and had already been used in the pilot study [25]. As with MAAP, NSSP consists of two 90-min group sessions per week over 7–10 weeks, plus one individual session to prepare for group therapy, resulting in a total of 14 sessions (1 individual and 13 group sessions). The scheduling flexibility is equally applied to both treatment arms and allows to reflect real-world clinical conditions (Fig. 1).

### Criteria for discontinuing or modifying allocated interventions {11b}

Criteria for discontinuing the allocated interventions include.

- Explicit participant request
- Discontinuation due to serious adverse event, including
  o Death of participant
  o Acute suicidality
  o Acute aggressiveness
  o Severe acute mental illness with indication for inpatient treatment lasting > 14 days

Patients with acute suicidality or serious aggressive behavior who require immediate crisis intervention are referred to an appropriate specialized facility, but can continue randomized treatment provided that the duration of the crisis intervention does not exceed 14 days. Outbreaks of violence during therapeutic sessions or life-threatening violence outside of therapeutic sessions lead to the termination of therapy. In this case, patients are referred to appropriate specialized treatment, with outpatient and inpatient services available.

### Strategies to improve adherence to interventions {11c}

Strategies to improve adherence on both the patient and therapist side include participation-related documentation completed by both the patient and therapist after each session, videotaping of therapy, and videotaped supervision by certified supervisors in the relevant treatment condition (each therapist must participate in supervision 3 times during an intervention period). Patient documentation includes the OAS-M (items 1–3) for overt aggression and the OAS-M (item 7) for suicidality, as well as questions about medication changes and use of psychiatric consultation. Therapist documentation includes assessment of acute suicidality and danger to others, as well as adherence to the treatment manual and interventions used.

### Relevant concomitant care permitted or prohibited during the trial {11d}

Relevant concomitant care that is prohibited during the trial (including follow-up period of 6 months).

- Other ongoing psychotherapy
- Increase in current psychotropic medication or initiation of new psychotropic medication within the last two weeks prior to randomization (for fluoxetine 6 instead of 2 weeks)
- Psychiatric care is permitted during the trial.

### Provisions for post-trial care {30}

Patients who discontinue therapy due to SAEs will continue to be managed according to good clinical practice until they are no longer an acute danger to themselves or others. It should be noted, however, that the risk associated with any type of group psychotherapy is low. In the pilot study, there were a total of two emergency hospitalizations; these are low in light of the high frequency of crisis hospitalizations typical of this patient population and were considered to be disorder-related rather than treatment-related. The occurrence of adverse events is not expected given the low level of psychological or physical distress associated with this study.

### Outcomes {12}

#### Primary outcome

Outward, overt aggression as measured by the first three items of the OAS-M 24 weeks after the end of treatment

#### Secondary outcomes

- Externally directed overt aggression in OAS-M (items 1–3) immediately after intervention and at follow-ups 4, 12, and 20 weeks after intervention.
- Attacks against self OAS-M (item 4), Irritability OAS-M (items 5–6) and suicidality OAS-M (item 7a/7b) at 24 weeks after intervention.
- OAS-M response rate (i.e., reduction in OAS-M sum of items 1–3 of at least 50% compared with baseline) at 24 weeks after intervention.
- Anger and aggressive behavior measured with Ecological Momentary Assessment (EMA) at 8 random time points in the 2 weeks before the 24-week follow-up (T5), adjusted for EMA at 8 random time points 2 weeks before the start of the intervention period.
- Symptom severity of BPD as measured by the Zanarini Rating Scale for Borderline Personality Disorder (ZAN-BPD) [31] at 24 weeks of follow-up.
- Anger as measured by the State-Trait Anger Expression Inventory (STAXI-II) [32] 24 weeks of follow-up.
- Everyday functioning as measured by the Social and Occupational Functioning Assessment Scale (SOFAS) [33] at 24 weeks of follow-up.
- Quality of life (WHOQoL-Bref) [34] at 24 weeks of follow-up.

#### Exploratory outcome

- Psychometric questionnaires used in exploratory analyses include German versions of the
  o Anger Rumination Scale (ARS) [35].
  o Personality Disorder Scale (PDS-ICD-11) [36].
  o Barratt Impulsiveness Scale (BIS-15) [37].
  o Brief-Symptom Checklist (BSCL-53) [38].
  o Social Network Questionnaire (SNQ) [39].
  o Personality Inventory for DSM-5 (PID-5-BF + M) [40].
  o UCLA Loneliness Scale (UCLA-3) [41].
  o Group Questionnaire (GQ-D) [42].
  o Negative Effects Questionnaire (NEQ) [43].
  o Difficulties in Emotion Regulation Scale (DERS-36) [44].
  o Certainty About Mental State Questionnaire (CAMSQ) [45].
  o Rejection Sensitivity Questionnaire (RSQ-20) [46].
- Computerized behavioral tasks used in exploratory analyses (assessed online at home) include
  o Emotion Classification Task [25] measuring attentional bias to threat-related stimuli by recording reaction times and error rates. Participants categorize 80 facial expressions (angry, fearful, happy, neutral) presented in a randomized order, following a fixation cross and brief stimulus display, with a response window of 3000 ms.
  o Grid Learning Aggression Task (GLAT; adapted from previous research on threat sensitivity) [47–49] where participants interact with several virtual agents on a grid to reach goals (similar to a PacMan game). Virtual agents can be cooperative or competitive, and the positions of the two players can be advantageous or disadvantageous for the participant. Participants rate their anger after the interaction.
  o Empathy and Theory of Mind (EmpaToM) [50] paradigm assessing empathy and Theory of Mind (ToM) abilities through a computer-based task involving short videos of individuals recounting autobiographical experiences with negative or neutral emotions. Participants rate their own emotional response to measure empathy and answer content-related questions, some requiring perspective-taking, to evaluate ToM abilities.

### Participant timeline {13}

#### Sample size {14}

In our pilot study with analogous study design and analogous patient population, the effect size for the primary outcome measure (OAS-M overt-aggression score at follow-up) was 0.68 (standardized mean difference between treatment arms on a log scale). For conservative planning of sample size, we assumed an effect size of 0.55. According to a sample size design with G*Power 3.1.9 [51], a sample size of 106 (53 per group) for a *t*-test with 80% power at a significance level of 0.05 with individual randomization would be indicated. In our pilot study, the intraclass correlation coefficient (ICC) within treatment groups (unit of randomization) was 0.075, which together with an average treatment group size of 4 corresponds to a variance inflation factor of 1.225. A sample size of 130 patients is therefore required for cluster randomization. With reference to previous studies and our pilot study [52, 53], a loss-to-follow-up of patients after randomization is expected with up to 30% of the randomized participants (drop-out 186*0.3 = 56), similar in both arms. This means that 186 patients will be randomized (93 to MAAP and 93 to the NSSP intervention) in order to observe the desired number of 130 patients with sufficient data for statistical analysis until the follow-up after 24 weeks. Our pilot study also indicates that a threefold number of patients than required should be screened for inclusion in the study. Against this background, a total of approximately 550 people should be screened for study eligibility. Based on our pilot study, 36% of the patients screened (by telephone) will not be included in the randomization for various reasons (e.g., not meeting the inclusion criteria, meeting the exclusion criteria, no longer available). Consequently, about 253 patients will be included in the diagnostic screening process at T-1, leaving 186 patients available for randomization. The sample to be recruited and examined thus comprises a total of *N* = 186 patients with (subthreshold) BPD and overt reactive aggression problems.

#### Recruitment {15}

Originally, recruitment at six sites in Germany (Aachen, Bielefeld, Bonn, Frankfurt, Heidelberg, Munich) was planned that cover metropolises as well as medium-sized cities that are more or less rural in character. To ensure sufficient recruitment within the planned trial phase, a seventh study site (Berlin) has been added after trial initiation. Various recruitment strategies are implemented based on our previous experience in the pilot study. Outpatients are recruited via psychiatric and/or psychotherapeutic specialist clinics for patients with BPD by providing information about the psychotherapeutic treatment available. Patients are also recruited via referrals from outpatient psychiatrists and psychotherapists, psychiatric clinics in the community as well as via the internet (study website, social media and networks), newspapers, radio, flyers, local anti-aggression projects, and registers of previous study participants in other trials. Existing collaborations with local experts by experience and self-help groups in several locations also contribute to patient recruitment.

## Assignment of interventions: allocation

### Sequence generation {16a}

Both the experimental and control interventions are implemented as group therapy, i.e., patients are randomized in clusters of therapy groups consisting of ideally 4 to 6 participants. Cluster randomization is performed using permuted block randomization, with a 1:1 allocation ratio to the two treatment arms. As soon as a group of usually 4 to 6 eligible participants at a given site has completed all baseline assessments, randomization of the group is performed. This process is initiated by a site staff member and conducted via a dedicated online platform managed centrally by the Coordination Center for Clinical Trials (KKS) in Heidelberg. This procedure ensures that allocation is both unbiased and concealed. Randomization is stratified by study site and must be completed prior to the start of the therapeutic sessions.

### Concealment mechanism {16b}

To ensure concealment of the randomization sequence until the time of intervention allocation, the allocation process is centrally managed by the KKS (allocation service team), which acts as a central, independent entity responsible for the allocation process and follows a set of robust procedures. After successful recruitment, treatment groups, each consisting of usually 4–6 participants, are randomly assigned to one of the two treatment arms in a 1:1 ratio while allocation is implemented digitally by designated study personnel at each study site using a software program that is accessible via a website. By centralizing randomization and concealing the allocation sequence until treatment group assignment, we ensure that the process remains unbiased.

### Implementation {16c}

After the successful recruitment of groups (4–6 patients) and the scheduling of assessments and therapy, group assignment is carried out by the responsible study coordinator at each site, or another dedicated member of the study staff who is not blinded to the assigned intervention (e.g., a research assistant) during the 2-week baseline assessment period.

## Assignment of interventions: blinding

### Who will be blinded {17a}

The outcome assessors (study diagnosticians) are blinded to the assigned intervention condition. Patients and all study personnel are informed and instructed not to disclose any information about the assigned intervention groups to the diagnosticians.

### Procedure for unblinding if needed {17b}

Participants and therapists cannot be blinded due to the nature of the intervention conditions. MAAP is a structured, mechanism-based psychotherapy that uses specific therapeutic techniques which differ substantially from the non-directive, supportive approach of NSSP. As such, participants are likely to recognize the type of therapy they are receiving, rendering patient blinding infeasible. However, participants are not informed about which intervention constitutes the control condition. This minimizes expectation effects and preserves some degree of blinding regarding the purpose of treatment allocation. Therefore, a formal unblinding procedure is not applicable.

## Data collection and management

### Plans for assessment and collection of outcomes {18a}

Table 1 provides a detailed overview of the assessments and collection of outcomes. During a personal or telephone screening (phone screening, T-2), interested patients are informed about the study and receive written information. General inclusion and exclusion criteria are also roughly checked via phone screening. At the next appointment (inclusion diagnostics, T-1), after informed consent has been signed, inclusion and exclusion criteria are checked in a detailed structured clinical diagnostic interview session, and comorbid disorders are also identified. The OAS-M [27] is used to assess the frequency and severity of overtly aggressive behavior. Severe cognitive impairments (IQ < 70) are screened using the mini-q [54] as well as clinical impression.

As soon as 4 to 6 patients have been included, they are invited to the next measurement appointment (baseline, T0), during which the OAS-M and the ZAN-BPD [55] are collected again. This baseline appointment must take place in the two weeks before the start of the intervention. At the beginning of T0, patients receive an online link to several psychometric questionnaires to assess the symptoms typical of BPD, the level of personality functioning, emotion regulation, everyday functioning, quality of life, sensitivity to rejection, anger rumination and experience, loneliness, social network, experiences of trauma and neglect in childhood and adolescence, general symptom burden and mentalization skills (for detailed overview, see Table 1). Patients also complete an Emotion Classification Task [25] and a Grid Learning Aggression Task (GLAT, adapted from [47–49]) to assess threat sensitivity as well as the EmpaToM [50], a paradigm independently manipulating empathy and ToM. Furthermore, EMA is completed during these two weeks before the start of the intervention.

During the 8- to 10-week intervention phase, the therapeutic alliance and adherence to therapy are measured repeatedly using questionnaires. The intervention phase is followed by the next measurement (T1), in which the OAS-M, the ZAN-BPD, and specific psychometric questionnaires from T0 (see Table 1) are collected again, and the emotion classification and GLAT tasks are also carried out again. At the follow-up measurements T2—T4 (4, 12 and 20 weeks after the end of the intervention), the OAS-M is collected again, and the psychometric questionnaires are administered in such a way that each of the questionnaires is collected once in total over the follow-up period (see Table 1). At the last measurement time point (T5, 24 weeks after the end of the intervention), all questionnaires (except from CTQ and LHA) and all behavioral tasks are completed again, while the EMA is also completed again during the two weeks prior to T5.

**Table 1:**
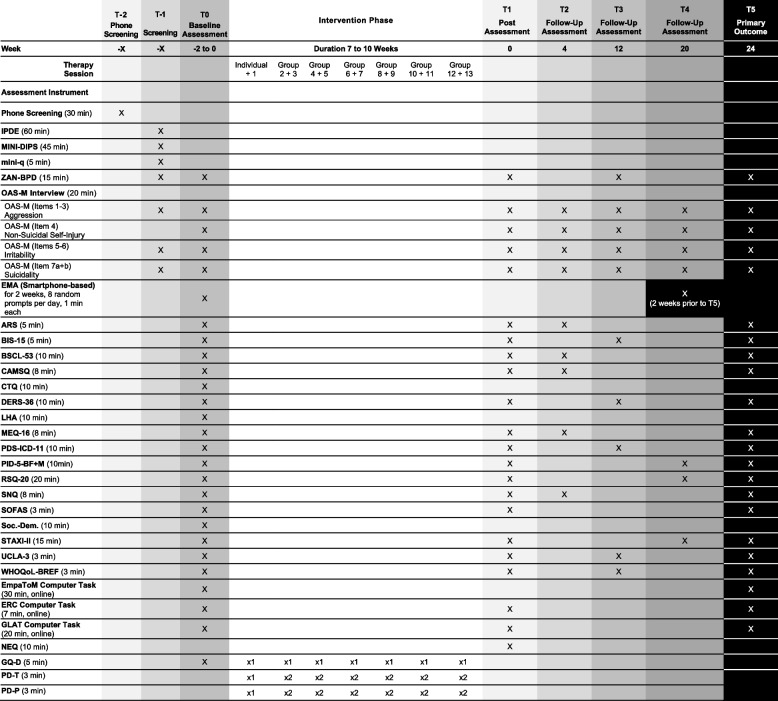
Overview of the study procedure and the assessments and collection of outcomes

IPDE (International Personality Disorder Examination for DMS-5): Suitability and assignment; MINI-DIPS for the assessment of exclusion criteria: Schizophrenia (lifetime), Bipolar I disorder (current), moderate and severe substance use disorder, and comorbidities; LHA (Life History of Aggression); mini-q: Assessment of verbal intelligence; ZAN-BPD (Zanarini Rating Scale for Borderline Personality Disorder): Measure of symptom severity; OAS-M (Overt Aggression Scale-modified): Interview-based measure of the frequency and severity of overt aggressive behavior that has occurred in a 2-week period; Items 1-3: verbal aggression, aggression against objects, aggression against others; Item 4: Aggression against oneself; Items 5–6: Irritability, subjective and visible from the outside; Suicidality: measured with item 7 and reviewed by a diagnostician with clinical expertise on an individual basis; ARS, Anger Rumination Scale; BIS-15, Barratt Impulsiveness Scale; BSCL-53, Brief-Symptom-Checklist; CAMSQ, Certainty About Mental States Questionnaire; CTQ, Childhood Trauma Questionnaire; DERS-36, Difficulties in Emotion Regulation Scale; EmpaToM, Empathy and Theory of Mind paradigm; ERC Emotion Classification task; GLAT Grid Learning Aggression Task; MEQ-16, Mentalizing Emotions Questionnaire; PDS-ICD-11, Personality Disorder Severity Scale; PD-T, Participation-related Documentation, Therapist Version; PD-P, Particpantion-related Documentation, Patient Version; PID-5-BF+M, Personality Inventory for DSM-5; RSQ-20, Rejection Sensitivity Questionnaire; SNQ, Social Network Questionnaire; SOFAS, Social and Occupational Functioning Assessment Scale; Soc.-Dem., Sociodemographic Questionnaire; STAXI-II, State-Trait-Anger-Inventory II; UCLA-3 Loneliness Assessment; WHOQoL-BREF, WHO Quality of Life; NEQ, Negative Events Questionnaire; GQ-D, Group Questionnaire, German Version

Note: Each shade corresponds to a specific phase or time point to help differentiate study phases over time. The assessment of the primary outcome is highlighted in black for easy identification

### Plans to promote participant retention and complete follow-up {18b}

In clinical studies of BPD, attrition rates of up to 30% have been reported after 6 to 12 months (e.g. [56],). Given the difficulty of follow-up in this patient population, we implement several strategies to promote participant retention and minimize missing data across all timepoints. Study personnel interacting with patients endeavor to establish and maintain communication and support through ongoing contact, which is likely to be a key factor in reducing dropout in this population. Regular contact is maintained with participants throughout the study period, including between treatment and follow-up assessments, using flexible and participant-preferred communication channels (e.g., via phone or email communication). Furthermore, follow-up assessments are scheduled with as much flexibility as possible to accommodate participants’ availability, including offering evening or remote appointments where appropriate. Participants are encouraged to complete outcome assessments even if they discontinue therapy, with dedicated research staff available to facilitate participation and resolve any barriers. Furthermore, participant reimbursement is structured to incentivize continued engagement: partial reimbursement is provided after completed assessments, with the final reimbursement amount also depending on the total number of questionnaires and EMA entries completed. This tiered approach is designed to reward ongoing participation throughout the study, aiming to reduce attrition and promote the completeness and quality of the data, particularly with regard to the primary outcome.

### Data management {19}

A dedicated data management team as part of the KKS ensures proper handling and storage of data throughout the study. This team ensures data integrity and compliance with data protection regulations. All data management processes and monitoring procedures are performed on validated systems in accordance with the current KKS SOPs and are defined in a study-specific monitoring manual (Investigator Site File, ISF). An electronic study-specific data collection form (electronic case report form, eCRF) managed by the KKS is used for data collection. The eCRF is used for data entry of all outcome measures, either directly (patient-administered psychometric questionnaires) or via paper–pencil post-collection (e.g., diagnostic interview sessions, patient and therapist documentation). All entries and corrections in the remote data collection system are automatically documented in an audit trail. The completeness, validity, and plausibility of the data are continuously checked and signed by authorized study personnel after data entry (edit checks) and with the help of validation programs that generate queries. Study participants are required to answer or clarify these queries. Once no further corrections are required, the database is closed and used for statistical analysis. At the end of the study, the data will be converted into various data formats (e.g., CSV files) to allow for reuse of the anonymized data.

### Confidentiality {27}

The names of patients and all other confidential information are subject to medical confidentiality and the provisions of the General Data Protection Regulation (GDPR) and the Federal Data Protection Act (*Bundesdatenschutzgesetz*, BDSG). Authorized representatives (persons responsible for data monitoring) who are obliged to maintain confidentiality may inspect the personal data held by the investigator insofar as this is necessary to verify the proper conduct of the study. With regard to this measure, the participants release the investigator from medical confidentiality. Other persons are not given access to original documents. Patient data may only be passed on in pseudonymized form.

### Plans for collection, laboratory evaluation and storage of biological specimens for genetic or molecular analysis in this trial/future use {33}

Not applicable, no biological specimens are collected or analyzed in this trial.

## Statistical methods

### Statistical methods for primary and secondary outcomes {20a}

Despite the cluster-randomized design, all outcomes are measured and assessed at the individual patient level. The expected superiority of MAAP over NSSP in reducing overt aggressive behavior is measured by the Overt Aggression Score (OAS-M, sum of items 1–3) at 24 weeks (primary outcome measure, T5). The superiority of the MAAP arm over the NSSP arm will be tested (at a two-sided significance level of 5%) using a linear mixed-effects model with overt aggression score as the dependent variable (using data of all post-randomization timepoints), pretreatment overt aggression score as a covariate and center, treatment arm, and time point as fixed effects, and patient ID and treatment group as random effects. The contrast between treatment arms at T5 is the parameter of interest and will be reported by effect size (standardized mean difference) and p-values as well as group-wise estimated marginal means (emmeans), all with 95% confidence intervals (CIs). Contrasts for all other post-randomization timepoints will be presented by effect size and emmeans with 95% CIs. Analyses of continuous secondary outcome measures will be performed analogously to the primary outcome measure, while analyses for binary secondary endpoints will be performed using logistic mixed-effects models.

### Interim analyses {21b}

There are no formal discontinuation criteria or stopping rules for the overall trial, as no serious health risks are expected. The intervention is considered low-risk, and similar study designs conducted by our group in the past have not resulted in any complications. In particular, a previous feasibility trial using the same interventions and procedures did not raise any safety concerns. Accordingly, no interim analyses are planned. This trial is intended to run to completion unless unexpected safety concerns arise.

### Methods for additional analyses (e.g. subgroup analyses) {20b}

Exploratory outcomes and endpoints for mediation analyses will be analyzed analogous to the primary outcome for continuous variables and using logistic mixed-effects models for binary variables. Safety data will be analyzed descriptively with lists of (S)AEs by treatment arm. To date, to the best of our knowledge, there is no evidence of gender-specific treatment effects in BPD. As men with BPD have often been neglected in BPD research [21], we plan an exploratory subgroup analysis by gender.

Data not relating to primary or secondary outcomes (e.g., baseline patient characteristics, safety data, or therapist adherence data) may be analyzed and published before the study is completed, as long as these analyses do not affect the interpretation of the primary or secondary outcomes. Such early analysis will be conducted with appropriate caution to ensure that it does not interfere with the integrity or the statistical power of the final results.

### Methods in analysis to handle protocol non-adherence and any statistical methods to handle missing data {20c}

The primary analysis will follow a modified intention-to-treat (mITT) approach, i.e., we will not include patients who do not participate in any of the randomized treatments (drop-out between eligibility and randomization, i.e., before the start of treatment). In general, the mixed-effects model uses all post-randomization measurements of the outcome of interest to test the treatment contrast at the last follow-up time point and thus effectively accounts for missing data. Only patients without any measurement except the baseline measurement do not contribute any information to this analysis. To investigate the potential impact of missing data, different sensitivity analyses are planned. Based on findings from the pilot study—where slightly more participants in the NSSP arm failed to complete T5—we do not assume that missing data are missing completely at random (MCAR), but rather potentially missing not at random (MNAR). Thus, two complementary multiple imputation strategies will be performed as sensitivity analyses. In the first multiple imputation strategy, imputation of missing outcome values at T5 will be performed separately for each treatment arm. In the second multiple imputation strategy, missing data will be imputed together for both treatment arms. A further sensitivity analysis will be carrying forward the last available overt aggression score from earlier timepoints (e.g., T4) to estimate T5 (LOCF).

Additionally, a per-protocol analysis will be performed as a further sensitivity check. This analysis will include only those participants who attended at least 9 out of 13 group sessions and did not miss more than 2 sessions in a row. This criterion is intended to ensure that only patients with adequate exposure to the assigned intervention are included in the per-protocol analysis.

### Plans to give access to the full protocol, participant level-data and statistical code {31c}

Due to the use of privacy sensitive information, the database will not be made available in a public repository. Anonymized participant-level data may be shared on reasonable request, e.g. for meta-analysis. The statistical code associated with peer-reviewed publications resulting from this study will be made available to the public.

## Oversight and monitoring

### Composition of the coordinating center and trial steering committee {5d}

This multicenter study was designed at the University Hospital of Heidelberg, Germany, and conducted at sites in Aachen, Berlin, Bielefeld, Bonn, Frankfurt, Heidelberg, and Munich. The coordinating center at the University Hospital Heidelberg is responsible for the overall management and organization of the study. Day-to-day support is provided by the sponsor/chief principal investigator, who maintains oversight of the trial, and the central study coordinator, who ensures the day-to-day administration and execution of the study protocol. This includes coordinating recruitment, data collection, and communication with and between sites.

The central study coordinator ensures that all trial-related tasks are in accordance with the protocol and that site-specific issues are addressed in a timely manner. Each participating site has one or two principal investigators, a study site coordinator, and additional dedicated study staff—including study therapists, diagnosticians, and research assistants—who play a key role in the conduct and day-to-day support of the trial.

The site study coordinators are responsible for the local execution of the study protocol, for patients’ safety and report of (serious) adverse events, overseeing activities such as patient recruitment, diagnostic assessments, treatment allocation, treatment delivery, follow-up visits, and other site-specific trial operations. Regular meetings are held both at the site level and across sites to maintain oversight, ensure protocol adherence, monitor safety, and support the achievement of study objectives.

Specifically, the project coordinators meet monthly to discuss trial progress, address protocol-related questions, and review recruitment, retention, and data quality across centers. The diagnosticians meet on a quarterly basis to ensure consistency in diagnostic procedures across sites, address assessment-related questions, and discuss any diagnostic challenges that may arise. In addition, all participating sites—including the principal investigators and the chief investigator—convene quarterly to review overall study progress, ensure protocol adherence, discuss site-specific developments, and coordinate cross-site collaboration and oversight.

### Composition of the data monitoring committee, its role and reporting structure {21a}

This trial has a Data Safety Monitoring Board (DSMB), an independent group of experts in psychiatry, clinical research, and patient safety who are responsible for ensuring patient safety throughout the trial. The primary responsibility of the DSMB is to ensure that the trial is conducted in accordance with the protocol, applicable regulatory requirements, and ethical guidelines, and that the safety of the participants is protected. The DSMB monitors (serious) adverse events (AEs and SAEs) and ensures that the trial is conducted according to safety standards. It provides strategic guidance on the progress of the trial, addresses any issues that arise during the trial, and makes recommendations for adjustments as necessary. The DSMB meets semi-annually with the sponsor and the central study coordinator to review safety data and recruitment and to make recommendations. In addition, the KKS provides data management support for this trial and oversees the scientific and ethical integrity of the trial. KKS monitoring is conducted at study initiation and annually at each participating site to ensure that the trial is following the protocol and regulatory requirements and to review progress, data safety and any emerging concerns.

### Adverse event reporting and harms {22}

There are no direct risks to patients participating in this study. All patients and one of the two group therapists fill out a participation-related documentation after each therapy session, including questions about acute suicidality. Patients also have the option of a brief crisis intervention with one of the two group therapists. Potential SAEs are defined as patient death, acute mental illness requiring hospitalization, acute suicidality, and acute aggression. Potential AEs are defined as delinquency or threat of violence, serious physical illness, serious accident, and new-onset acute mental disorder (comorbid with borderline personality disorder). The study collects, assesses, and manages AEs and SAEs, as well as any unintended effects related to the study interventions or their administration. AEs and SAEs are recorded; SAEs are reported to the chief principal investigator within 24 h, and then reported to the DSMB. To this end, the occurrence of SAEs is categorized, and their severity, relationship to study interventions, and outcome are systematically assessed and documented in the eCRF. Appropriate follow-up actions will be taken for all SAEs in consultation with the DSMB. The DSMB will advise whether the trial should be continued or stopped if a cluster of SAEs is suspected. These decisions will be based on ongoing risk assessments and careful evaluation of the available safety data. Periodic reviews are performed and results are reported to the ethics committees as required.

Any patient showing signs of acute suicidality or severe aggression requiring immediate crisis intervention will be referred to specialized facilities while continuing their randomized treatment, provided that the crisis intervention does not exceed 14 days. In cases of violence, either during therapy sessions or in life-threatening circumstances outside of therapy, therapy will be discontinued and the participant will be referred to appropriate care, either inpatient or outpatient.

### Frequency and plans for auditing trial conduct {23}

In accordance with Good Clinical Practice (GCP) standards, trial conduct is subject to multiple levels of monitoring and oversight to ensure compliance with the protocol, regulatory requirements, and ethical guidelines. The Trial Steering Committee and the DSMB (representing the independent Data Monitoring Committee) convene every six months to evaluate the overall conduct of the trial, review accumulated data, and monitor for any potential safety or protocol adherence issues. In addition, independent monitoring is conducted annually by the KKS. These audits are performed in accordance with ICH-GCP guidelines and the KKS Standard Operating Procedures (SOPs). The KKS conducts on-site visits once per year at each site, with the option for remote video monitoring if needed. These audits cover documentation of informed consent, participant data, administration of interventions, and reporting of (S)AEs. All monitoring reports are subject to quality control by the KKS and are forwarded to the coordinating site for review and, if necessary, action. Given the low-risk nature of the intervention, no additional ethics committee meetings are conducted beyond the initial ethics approval and oversight provided by the institutional review boards. The combination of DSMB oversight and KKS monitoring ensures that participant safety, data integrity, and regulatory compliance are maintained throughout the trial.

### Plans for communicating important protocol amendments to relevant parties (e.g. trial participants, ethical committees) {25}

The protocol and any subsequent amendments have been and will continue to be submitted to the University of Heidelberg Ethics Committee for review and approval to ensure that any changes to the study are ethically acceptable. In the event of a protocol amendment, the sponsor and funder will be notified first. Following their approval, the coordinating investigator will inform all participating centers. The principal investigator at each site will receive the updated version of the protocol, which is also added to the ISF. Any major changes to the protocol (e.g., in recruitment, assessment and outcome collection, or data management) are promptly communicated to all relevant parties by the coordinating site. Notifications occur through updated versions of the protocol sent via email and/or communicated in telephone or videoconference meetings. Changes that impact participant safety, study design, or ethical considerations will be submitted to the responsible ethics committee at each site for approval prior to implementation. Participants will be informed of any modifications affecting their participation, and updated informed consent forms will be provided if required. In addition, all protocol amendments will be reflected in the trial registry to ensure public transparency. Any protocol deviations will be fully documented using a special online form embedded in the eCRF. In cases where substantial changes occur, these will be reported to regulatory authorities and relevant journals in accordance with regulatory and publication standards. All changes are documented and required approvals or notifications are obtained prior to implementation.

## Dissemination plans {31a}

Results will be published in peer-reviewed journals and at research conferences. Important protocol modifications will be reported at the trial registry German Clinical Trials Register DRKS (DRKS00031608). Due to the use of privacy-sensitive information, the database will not be made available in a public repository.

### Patient and public involvement

Patients and individuals with lived experience of BPD were actively involved in the development of the treatment program and the design of the present clinical trial. Specifically, patient advocates and experience experts participated in the conceptualization of intervention components and contributed feedback on study procedures to enhance acceptability, safety, and relevance. Their contributions informed both the therapeutic content and the trial design. While patients are not involved in the day-to-day trial management, their early involvement ensured that the study aligns with the principles of patient-centered research and enhances the clinical relevance of the findings.

## Discussion

This protocol presents a large-scale, multicenter RCT aimed at assessing the efficacy of a mechanism-based anti-aggression group psychotherapy program for individuals with BPD [57]. The trial is designed to evaluate the superiority of MAAP over a non-specific supportive psychotherapy (NSSP) control intervention [58] in reducing outward-directed aggression, a key feature of BPD.

Overt aggression is a common, functionally debilitating, and clinically challenging aspect of BPD. Studies have highlighted the occurrence of reactive aggression triggered by interpersonal stressors such as perceived rejection or threat [10, 59–61]. However, despite the substantial burden that aggression imposes on individuals and their social environments, effective and cost-efficient treatment options remain limited. This trial seeks to address this gap by investigating a psychotherapeutic approach targeting the mechanisms underlying outward-directed aggression in BPD, such as social threat hypersensitivity, approaching social threat cues rather than avoiding them, maladaptive anger regulation, excessive emotional imitation and contagion, and difficulty mentalizing others’ intentions, cognitions, and emotions adequately [57]. The mechanisms-based design of MAAP has the potential to directly address these core factors, paving the way for more tailored interventions in BPD.

The results of this trial will provide valuable insights into the broader impact of MAAP on BPD symptoms beyond aggression. Secondary outcomes, including self-harm behavior, irritability, anger experience, and improvements in everyday functioning and quality of life, will help establish whether MAAP not only addresses aggression but also improves general functioning and well-being. These findings would be critical in demonstrating the broader benefits of MAAP as a treatment for BPD, a disorder historically difficult to treat. Furthermore, the study may contribute to the development of mechanism-based psychotherapies in general.

Several limitations and challenges are associated with this trial. Conducting a study of this scale and complexity presents practical and operational challenges that require proactive planning, strict adherence to protocols, and flexibility in adapting to unforeseen issues. Recruitment and retention of participants are likely to be key concerns. The specific diagnostic criteria for BPD, combined with the need to identify patients with clinically significant aggression, will limit the pool of eligible participants. Additionally, willingness to commit to repeated assessments over an extended period may influence enrollment and retention of patients. Although compensation and support are in place, some patients may drop out for personal, logistical, or psychological reasons. Ensuring patient engagement and minimizing dropout rates will be of especially high importance in this trial. Strategies such as flexible follow-up options, including virtual consultations, and consistent communication will help address these challenges. Data management also presents significant operational challenges. With multiple study sites involved, discrepancies in data collection or reporting could affect the validity of the findings. The use of an eCRF and validation checks will help to minimize these issues, but challenges (e.g., challenges concerning accurate and timely data entry) may persist. Missing or incomplete data will require statistical strategies to maintain robust results. Furthermore, adherence to the study protocol is essential for the reliability of outcomes. Any deviation, whether due to participant non-compliance or inconsistencies in study staff implementation, could introduce bias. Variability across sites may generally impact recruitment, retention, and data collection. Differences in site staff, infrastructure, and resources could affect protocol fidelity and the quality of data. To address these challenges, we organize regular meetings of diagnosticians, study coordinators, and PIs to discuss specific questions, uncertainties, or other difficulties and to develop solutions. Monitoring mechanisms, such as site visits, remote oversight, and strong orientation towards GCP guidelines, will minimize these risks, though deviations remain a possibility. Clear communication with and between sites and ongoing training is crucial for ensuring consistent protocol adherence across all study sites. Lastly, given that participants may be at risk for severe adverse events, including suicidality and aggression, managing participant safety will remain a priority. The study includes safety measures like a DSMB and crisis intervention protocols, but unforeseen safety issues may arise, requiring rapid responses and potential protocol adjustments. These concerns highlight the importance of maintaining flexibility and short-term responsiveness in trial management.

In summary, and to the best of our knowledge, this trial is the first to specifically target outward-directed aggression in BPD using a mechanism-based psychotherapy approach. The results of this study are critical in addressing a significant gap in the treatment of aggression in BPD, as existing interventions focus predominantly on self-directed aggression, such as self-harm and suicidal behavior. The focus on outward-directed aggression could offer a more targeted and effective treatment approach, which is currently lacking in clinical practice. Thus, this study represents an important step toward improving the treatment of aggression in BPD. By focusing on the biobehavioral and emotional processes underlying reactive aggression, MAAP offers a novel intervention that could substantially change the therapeutic landscape for BPD. While the trial design addresses many of the common challenges in clinical research, such as recruitment, data management, and safety monitoring, flexibility will be essential to adapt to unanticipated challenges. If successful, this trial will not only advance the scientific understanding of aggression in BPD, but also pave the way for more targeted and effective interventions for this challenging population, which will also be more cost-effective given a group therapeutic approach, another important consideration given the enormous economic burden of BPD, including high direct and indirect costs of illness. The results of this trial could have broad clinical implications, potentially leading to the widespread adoption of MAAP in psychiatric care and offering a new approach to treating one of the most disruptive and debilitating symptoms of BPD.

### Trial status

The current protocol version is 1.0, dated March 7, 2025. The first patient was enrolled in May 2024, and the planned date for the last patient out is November 2026.

## Data Availability

Due to limits of consent for data sharing and the use of privacy sensitive information, datasets cannot be provided to external parties and will not be published on a public repository.

## Abbreviations

AE: Adverse event
BDSG: Federal Data Protection Act (*Bundesdatenschutzgesetz*)
BPD: Borderline personality disorder
CSV: Comma-separated values
DBT: Dialectical behavior therapy
DFG: German Research Foundation (*Deutsche Forschungsgemeinschaft)*
DMC: Data Monitoring Committee
DRKS: German Registry for Clinical Studies (*Deutsches Register Klinischer Studien*)
DSM(-5): Diagnostic and Statistical Manual of Mental Disorders (Fifth Edition)
DSMB: Data Safety Monitoring Board
eCRF: Electronical Case Report Form
EMA: Ecological Momentary Assessment
GCP: Good clinical practice
GDPR: General Data Protection Regulation
ICC: Intraclass correlation coefficient
ICD: International Statistical Classification of Diseases and Related Health Problems
IPD: Individual patient data
IPDE: International Personality Disorder Examination
ISF: Investigator site file
KKS: Coordination Center for Clinical Trials (*Koordinierungszentrum für Klinische Studien*)
MAAP: Mechanism-based anti-aggression psychotherapy
MBT: Mentalization-based therapy
mITT: Modified Intention-to-treat
NSSP: Non-specific supportive psychotherapy
PI: Principal Investigator
RCT: Randomized controlled trial
SAE: Serious adverse event
SOP: Standard operating procedure
TAU: Treatment as usual
